# High-cholesterol diet enriched with onion affects endothelium-dependent relaxation and NADPH oxidase activity in mesenteric microvessels from Wistar rats

**DOI:** 10.1186/1743-7075-11-57

**Published:** 2014-12-23

**Authors:** Diana González-Peña, Javier Angulo, Susana Vallejo, Clara Colina-Coca, Begoña de Ancos, Carlos F Sánchez-Ferrer, Concepción Peiró, Concepción Sánchez-Moreno

**Affiliations:** Institute of Food Science, Technology and Nutrition (ICTAN), Spanish National Research Council (CSIC), José Antonio Novais 10, ES-28040 Madrid, Spain; Servicio de Histología-Investigación, Instituto Ramón y Cajal de Investigación Sanitaria (IRYCIS), ES-28034 Madrid, Spain; Departamento de Farmacología, Facultad de Medicina, Universidad Autónoma de Madrid, ES-28029 Madrid, Spain

**Keywords:** Onion, Functional foods, Dietary cholesterol, Mesenteric microvessels, NADPH oxidase

## Abstract

**Background:**

The aim of the present study was to examine the effects of onion as functional ingredient on the oxidative status, lipoprotein levels (total cholesterol-TC, HDL-C, LDL-C), triacylglycerides (TAG) and vascular reactivity of mesenteric arteries in hypercholesterolemic Wistar rats.

**Methods:**

Twenty-four animals were fed with three different diets [control, high-cholesterol diet (HC) and high-cholesterol enriched with onion diet (HCO)]. After seven weeks of experimental feeding the rats were euthanized for blood and tissues collection. TC, HDL-C, LDL-C and TAG were measured, and 2,2'-azinobis-(3-ethylbenzothiazoline-6-sulfonic acid) radical cation (ABTS^•+^) scavenging capacity and ferric reducing antioxidant power (FRAP) were determined in plasma. Superoxide dismutase (SOD) and glutathione peroxidase (GPx) enzyme activities were assayed in erythrocyte lysates. Endothelium-dependent vasodilation to acetylcholine was evaluated in mesenteric arterial segments. NADPH oxidase (NOX) was also measured by lucigenin-derived chemiluminiscence.

**Results:**

The dietary cholesterol content significantly affected plasma lipoprotein levels, increased superoxide generation from NOX, and caused impaired endothelium-dependent vasodilation in the rat mesenteric arteries. Onion ingredient improved antioxidant status in HCO group, as it was evidenced by ABTS^•+^ and FRAP values and SOD and GPx enzyme activities compared to the HC-fed group, reduced the increment in NOX activity and reversed endothelial dysfunction promoted by the HC diet. Scavenging of superoxide with TEMPOL or inhibition of NOX with apocynin improved endothelium-dependent vasodilation only in HC-fed rats.

**Conclusions:**

Enrichment of diet with onion as functional ingredient could be proposed as a complementary approach to prevent or partially modulate vascular dysfunction, reducing some of the risk indexes linked to initial development of atherosclerosis.

## Background

The evolution of hypercholesterolemia is associated with a large range of alterations considered as strong risk factors for many negative cardiovascular events. Elevated oxidant stress linked to pro-inflammatory conditions contributes to the development of alterations in the bioavailability of vascular nitric oxide and some endothelial cell dysfunctions that can culminate in profound impairments to vascular reactivity [1]. The redox status linked to hypercholesterolemic conditions is affected by an imbalance between prooxidant and antioxidant enzymes, where NADPH oxidases (NOX), xanthine oxidase, and myeloperoxidase could be pointed as the major oxidant producing systems which lead to an overproduction of free radicals. In addition, some of the markers of oxidative stress might be used as a tool to predict cardiovascular risk, since its upregulation has been usually associated to inflammatory responses within the microvasculature [2, 3].

The vascular endothelium is a multifunctional organ system composed of metabolically active and physiologically responsive component cells that regulate blood flow depending on metabolic conditions. The sensitivity of such system is able to display evidences of endothelial dysfunction, like one of the earliest events in the development of atherosclerosis [4]. Dietary intake of certain nutrients has been suggested as an alternative in the prevention of several pathologies, as a gentle mean of modifying the mechanisms involved in the progression of cardiovascular disease [5–9]. Recent evidences in this line have pointed out the consumption of flavonoid-rich food such as wine and grape related products, chocolate and soya in the improvement of different endothelial functions [10–12], the consumption of the well-known rich in antioxidants Mediterranean diet in the amelioration of endothelial vasodilation in hypercholesterolemic subjects [13] and the decrease in the incidence of major cardiovascular events in persons with cardiovascular risk [14].

In this sense, the potential efficacy of onion consumption in the prevention of vascular damage have been highly supported by its hypolipidemic, antiinflamatory, antihypertensive, antidiabetic, antithrombotic, and antihyperhomocysteinemia effects [15, 16]. Specific phytochemicals (flavonols, sulphur compounds) and nutritional compounds (dietary fibre) present on its matrix have been pointed out as main promoters of those cardioprotective effects [17–19]. However, the advantage of consuming the natural product instead of those separated compounds must be taken in consideration since interactions and synergistic effects shall be present and responsible for some specific health benefits. In this regard, a branch of the food processing field has focused its efforts in facilitating ready-to-use functional ingredients that preserve (or even improve) the quality, content of bioactive compounds, and the functional activities of the original raw material. The enhancement of some compounds present in onion, and their stabilization by using combined technologies have made available an stable functional food ingredient with great versatility and added value due to its potential biological activity [20].

The application of this so-called "functional ingredient” arises as a possible intervention for either the improvement of vascular function or as an alternative strategy in the prevention of the development of cardiovascular disease.

Consequently, the aim of the present study was to investigate the effects of onion as functional ingredient on lipid profile, oxidative status, and endothelium-dependent relaxation and NOX activity in mesenteric microvessels in hypercholesterolemic Wistar rats.

## Methods

### Onion powder preparation

Raw onions (*Allium cepa* L. var *cepa*, ‘Recas’) were supplied by Cebacat (Asociación Catalana de Productores-Comercializadores de Cebolla, Lleida, Spain). Onions were harvested in April 2012 in Spain and their bulbs were free of external damages and stored at 4°C until processing (5 days later). The onions were hand-peeled, cut into 10 mm pieces, packaged in bags with very low gas permeability (Doypack®, Polyskin XL, Amcor Flexibles Hispania, S.L., Granollers, Barcelona, Spain) and treated by high-pressure. Diced packed onions were exposed to 400 MPa for a 5-min hold-time and processing unit temperature at 25°C. After the high-pressure treatment, the onion was immediately frozen with liquid nitrogen, freeze-dried in a lyophilizer (model Lyoalfa, Telstar, S.A., Barcelona, Spain), pulverized with a ultra centrifugal mill ZM 200 (Retsch GmbH, Haan, Germany) obtaining a fine powder (final size particle ≤ 250 μm), and stored at -20 ± 0.5°C until use. Nutritional composition, phytochemical compounds, and antioxidant activity of onion powder are shown in Table 1.

**Table 1:** **Nutritional composition, phytochemical compounds, and antioxidant activity of onion powder**

	Onion powder
Protein (g/100 g)	9.75 ± 0.08
Lipids (g/100 g)	1.30 ± 0.06
Carbohydrates (g/100 g)	80.10 ± 2.89
Glucose (g/100 g)	27.7 ± 1.73
Fructose (g/100 g)	20.7 ± 0.46
Sucrose (g/100 g)	4.3 ± 0.11
Total fructans (g/100 g)	4.2 ± 0.10
Total dietary fibre (g/100 g)	23.2 ± 1.15
Soluble fibre (g/100 g)	3.2 ± 0.09
Insoluble fibre (g/100 g)	20.0 ± 0.17
Ash (g/100 g)	4.63 ± 0.07
Total phenols (mg GAE/100 g)	1629.6 ± 60.0
Quercetin 3-glucoside (mg/100 g)	32.22 ± 0.90
Quercetin 4'-glucoside (mg/100 g)	950.00 ± 2.99
Quercetin 3,4'-diglucoside (mg/100 g)	1368.89 ± 8.77
Quercetin 7,4'-diglucoside (mg/100 g)	31.56 ± 0.33
Quercetin 3,7,4'-triglucoside (mg/100 g)	9.16 ± 0.32
Isorhamnetin 4'-glucoside (mg/100 g)	45.16 ± 1.54
Isorhamnetin 3,4'-diglucoside (mg/100 g)	32.00 ± 0.19
Total ACSOs (mg BCSOE/100 g)	4120.89 ± 89.43
Propionaldehyde (mg/100 g)	245.04 ± 39.61
1-Propanethiol (mg/100 g)	23.54 ± 0.90
Hexanal (mg/100 g)	0.04 ± 0.001
2-Methyl 2-pentenal (mg/100 g)	10.80 ± 0.67
Propyl thioacetate (mg/100 g)	0.45 ± 0.03
Dimethyl trisulfide (mg/100 g)	66.41 ± 5.02
Dipropyl disulfide (mg/100 g)	89.45 ± 3.29
Methyl propyl trisulfide (mg/100 g)	42.28 ± 2.14
Dipropyl trisulfide (mg/100 g)	25.50 ± 2.45
Ascorbic acid (mg/100 g)	62.31 ± 0.77
Total vitamin C (mg/100 g)	104.26 ± 4.07
Scavenging of NO^•^ (μmol TE/100 g)	1706.00 ± 49.61
ABTS^•+^ (μmol TE/100 g)	4936.67 ± 72.65
DPPH^•^ (μmol TE/100 g)	1135.00 ± 82.21
FRAP (μmol TE/100 g)	12245.14 ± 60.45

Values are expressed as the mean ± SD (n = 3).

GAE, gallic acid equivalents; ACSOs, *S*-alk(en)yl-L-cysteine sulfoxide; BCSOE, *S*-Butyl-L-cysteine sulfoxide equivalents; NO^•^, nitric oxide radical ABTS^•+^, 2,2′-azinobis(3-ethylbenzothiazoline-6-sulfonic acid) radical cation; DPPH^•^, 2,2-diphenyl-1-picrylhydrazyl radical; FRAP, ferric reducing antioxidant power; TE, trolox equivalents.

### Diet preparation and experimental design

A total of twenty four male Wistar rats with a body weight of approximately 250 g at the outset were obtained from Harlan Laboratories Models (Harlan, SL, Barcelona, Spain). The animals were housed individually in metabolic cages in a temperature-controlled room (22.5 ± 0.5°C) with a 12 h light − 12 h dark cycle. The present study was approved by the Spanish Ministry of Science and Innovation Advisory Committee [project AGL2010-15910 (subprogram ALI)] and by an Ethics Committee of the Complutense University of Madrid (Spain). All experiments were performed in compliance with the Directive 2010/63/UE regarding the protection of animals used for scientific purposes. The rats were fed commercial rat pellets (Panlab, SLU, Barcelona, Spain) for 3 days for the adaptation to environmental conditions and then distributed into three groups of eight animals each, according to average body weight and fed the control diet for 4 days for the adaptation to the metabolic cages. The diets composition, based on the AIN-93 M semi-purified rodent diet [21], is shown in Table 2. The following three experimental semi-synthetic diets (Table 2) were prepared: (1) the control diet was composed of a homogeneous mixture of 100% rodent diet; (2) the high-cholesterol diet (HC) was the control diet with 2% cholesterol and 0.5% cholic acid, substituting an equal amount of maize starch; and (3) the high-cholesterol diet enriched with onion (HCO) was identical to the high-cholesterol diet, but with 10% onion powder, balancing the dietary fibre with cellulose powder. The dose for onion powder was selected based on the body surface area normalization method and previous studies [22, 23]. Water and food were provided *ad libitum* over the 7-week experimental period. Body weight and food consumption were recorded weekly and daily, respectively.

**Table 2:** **Composition of the experimental diets‡**

Ingredient (g/kg)	Control	HC	HCO
Onion powder	−	−	100
Casein	200	200	200
Sucrose	100	100	100
Maize starch	470.49	445.49	368.69
Soya oil	50	50	50
Maize oil	80	80	80
Mineral mixture*	35	35	35
Vitamin mixture†	10	10	10
Cellulose powder	50	50	26.8
Choline bitartrate	2.5	2.5	2.5
*tert*-butylhydroquinone	0.010	0.010	0.010
L-cystine	2	2	2
Cholesterol	−	20	20
Cholic acid	−	5	5

HC, high-cholesterol diet; HCO, high-cholesterol enriched with onion diet.

*Mineral mix for the AIN-93 M diet, g/kg: calcium carbonate anhydrous, 357.00; potassium phosphate monobasic, 250.00; potassium citrate, tripotassium monohydrate, 28.00; sodium chloride, 74.00; potassium sulphate, 46.00; magnesium oxide, 24.00; ferric citrate, 6.06; zinc carbonate, 1.65; sodium meta-silicate 9H_2_O, 1.45; manganous carbonate, 0.63; cupric carbonate, 0.30; chromium potassium sulfate 12H_2_O, 0.275; boric acid, 0.0815; sodium fluoride, 0.0635; nickel carbonate, 0.0318; lithium chloride, 0.0174; sodium selenate anhydrous, 0.01025; potassium iodate, 0.0100; ammonium paramolybdate 4H_2_O, 0.00795; ammonium vanadate, 0.0066; powdered sucrose, 209.806.

^†^AIN-93-VX vitamin mix for the AIN-93 M diet, g/kg: niacin, 3.000; calcium pantothenate, 1.600; pyridoxine-HCl, 0.700; thiamin-HCl, 0.600; riboflavin, 0.600; folic acid, 0.200; biotin, 0.200; vitamin B12 (0.1%), 2.500; vitamin E (all-*rac*-α-tocopheryl acetate, 500 IU/g), 15.000; vitamin A (all-*trans*-retinyl palmitate, 500,000 IU/g), 0.800; vitamin D3 (400,000 IU/g), 0.250; vitamin K1, 0.075; powdered sucrose, 974.655.

‡Diet energy content was calculated using the factors 16.73 kJ/g (4 kcal/g) for protein, 15.69 kJ/g (3.75 kcal/g) for monosaccharides, 16.53 kJ/g (3.95 kcal/g) for disaccharides, 17.49 kJ/g (4.18 kcal/g) for starch, 8.37 kJ/g (2 kcal/g) for dietary fibre, and 37.65 kJ/g for fat. Control diet, 18540.9 kJ/kg (4431.4 kcal/kg); HC diet, 18856.6 kJ/kg (4506.8 kcal/kg); HCO diet, 18642.4 kJ/kg (4455.6 kcal/kg).

### Sampling

At the end of the experiment, in order to avoid inter-assay variations that could affect the comparison of data from the different groups, animals in fasting conditions were anaesthetised and euthanized by extracting blood by cardiac puncture with a syringe, taking one animal at a time, of each one of three groups. Blood was collected from the heart and taken into tubes with EDTA as anticoagulant. Plasma was recovered after centrifugation (1500 *g*, 15 min) at 4°C and immediately stored at −80°C until analysis. Third branch mesenteric arteries (lumen diameter 200–400 μm) were obtained from rat omentum specimens and placed into ice-cold Krebs-Henseleit solution (KHS; composition in mM: NaCl 119, KCl 4.6, CaCl_2_ 1.5, MgCl_2_ 1.2, NaHCO_3_ 24.9, glucose 11, KH_2_PO_4_ 1.2 and EDTA 0.027) and transported to the laboratory for dissection within 2 h of collection.

### Nutritional composition, phytochemical compounds, and antioxidant activity of onion powder

Analysis of protein, lipids, total fructans, total dietary fibre and ash was performed using standard laboratory procedures [24]. Soluble sugars were determined by ion chromatography using the method described by Colina-Coca *et al*. [25].

Determination of total phenolic content according to the Folin − Ciocalteu method, identification and quantification of flavonols by HPLC-DAD and HPLC-ESI-MS, determination of total *S*-alk(en)yl-L-cysteine sulfoxide (ACSOs) by HPLC-DAD, determination of volatile compounds by headspace GC-MS, and measurement of ascorbic acid and total vitamin C by HPLC-DAD were performed using the methods described by González-Peña *et al*. [20], and Colina-Coca *et al*. [25, 26].

Determination of nitric oxide radical (NO^•^) scavenging capacity, 2,2′-azinobis(3-ethylbenzothiazoline-6-sulfonic acid) radical cation (ABTS^•+^) scavenging capacity, 2,2-diphenyl-1-picrylhydrazyl radical (DPPH^•^) scavenging capacity, and ferric reducing antioxidant power (FRAP) were carried out using the methods described by González-Peña *et al*. [20].

### Plasma cholesterol and triacylglycerides analyses

The total cholesterol (TC), HDL-cholesterol (HDL-C), LDL-cholesterol (LDL-C) and triacylglycerides (TAG) were determined in the plasma using standard enzymatic colorimetric methods (SPINREACT, SA/SAU, Girona, Spain) using a COBAS INTEGRA 400 plus system (Roche Diagnostics Ltd., Rotkreuz, Switzerland). Atherogenic indexes (AI) were calculated as follows: AI (1) = LDL-C/HDL-C, AI (2) = TC/HDL-C, AI (3) = (TC-HDL-C)/HDL-C.

### Plasma antioxidant activity analyses

2,2′-azinobis(3-ethylbenzothiazoline- 6-sulfonic acid) radical cation (ABTS^•+^) scavenging capacity and ferric reducing antioxidant power (FRAP) were carried out in plasma using the methods described by González-Peña *et al*. [20] slightly modified.

### Erythrocyte antioxidant enzyme activities

Blood was treated to obtain the erythrocyte lysates as specified in the kits before assaying for SOD and GPx activities. SOD and GPx, activities were measured using a Superoxide Dismutase Assay Kit (706002, Cayman Chemical), and a Glutathione Peroxidase Assay Kit (703102, Cayman Chemical), respectively.

### Endothelium-dependent relaxation analyses in mesenteric microvessels. Vascular reactivity

Third branch mesenteric arteries were dissected by carefully removing the adhering fat tissue. Arterial ring segments (2 mm long) were subsequently mounted on microvascular wire myographs (J.P. Trading, Aarhus, Denmark) for isometric tension recordings as previously described [27, 28]. The vessels were allowed to equilibrate for 30 min in KHS continuously bubbled with 95% O_2_/5% CO_2_ mixture to maintain a pH of 7.4. Passive tension and internal circumference of vascular segments when relaxed *in situ* under a transmural pressure of 100 mmHg (L_100_) were determined. The arteries were then set to an internal circumference equivalent to 90% of L_100_, at which the force development was close to maximal [29]. The preparations were then exposed to 125 mM K^+^ (KKHS, equimolar substitution of NaCl for KCl in KHS) and the contractile response was measured. After a stabilization period, rat arteries were contracted with 1–3 μM noradrenaline (NA, 80% of KKHS induced contraction, approximately) and relaxation responses were evaluated by cumulative additions of acetylcholine (ACh; 1nM to 30 μM), to the chambers. Experiments were run in parallel. For determining the effects of superoxide scavenging and inhibition of NOX on endothelium dependent responses, arterial segments were incubated for 30 min with TEMPOL (10 μM) or apocynin (10 μM), respectively. Concentration-response curves to ACh in arterial segments from the same animal that previously received only vehicle (distilled water) were considered as controls for the evaluation of the effects of these treatments. For the evaluation of the impact of hypercholesterolemia on endothelium-independent vasodilatations, cumulative additions of sodium nitroprusside (SNP; 1 nM to 100 μM) were added on NA-precontracted arterial segments from control and HC-fed rats.

### NADPH oxidase activity in mesenteric microvessels

The activity of NADPH oxidase (NOX) was measured by lucigenin-derived chemiluminiscence [28]. Mesenteric arteries from control, HC-, and HCO-fed rats were snap frozen in liquid N_2_ and stored at −80°C until NOX activity assay. Arterial tissue was homogenized in lysis buffer (pH 7.0) containing 50 mmol/L KH_2_PO_4_, 1 mmol/L EGTA and 150 mmol/L sucrose for 5 min at 4°C. For every sample, the protein content was determined by the bicinchoninic acid method. Vascular extracts were incubated in phosphate-buffered saline containing 5 μmol/L lucigenin and 100 μmol/L NADPH and luminiscence was then measured every 30 s for 5 min in a microplate luminometer (Berthold Orion II, Titertek-Berthold, Pforzheim, Germany). The enzymatic activity was expressed as relative light units (RLU)/mg of protein/min.

### Statistical analyses

Results are expressed as mean values with their SD or SEM as appropriate. Data were analysed using one-way ANOVA. Levene’s test was applied to verify the homogeneity of the variances. Tamhane’s T2 (equal variances not assumed) and Bonferroni (equal variances assumed) *post hoc* tests were used to determine differences within groups with significance set at *P* < 0.05. Analyses were performed using the IBM SPSS Statistics 21 (SPSS Inc., an IBM Company). Relaxation responses are expressed as the percentage of the remaining noradrenaline-induced contraction. pD_2_ is defined as the -log M of the concentration required to obtain 50% of maximal relaxation. Complete concentration-response curves were obtained and compared by two-way ANOVA using StatView software for Apple computers (SAS, Cary, NC).

## Results

### Food intake and body weight gain

**Figure 1:**
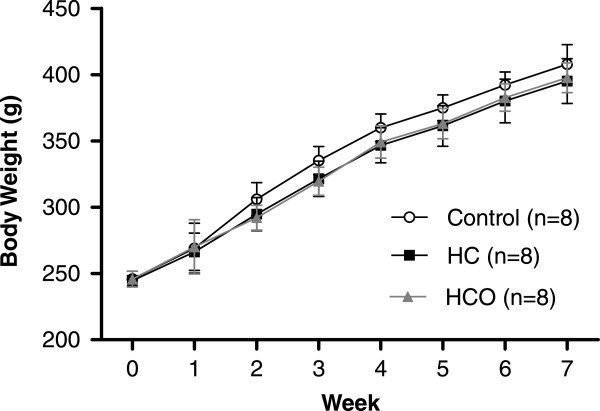
**Growth curve of rats fed control, high-cholesterol (HC) and high-cholesterol enriched with onion (HCO) diets for 7 weeks.** Data are expressed as the mean ± SD.

The food intake (g/day) of the rats did not differ among the groups during the feeding period (16.14 ± 0.20 for control, 15.99 ± 0.18 for HC, and 16.33 ± 0.26 for HCO). As the growth curve shows (Figure 1), there was no significant difference in the body weight gain (g) among the three groups after the feeding period (141.80 ± 9.75 for control, 133.68 ± 6.93 for HC, and 132.00 ± 9.36 for HCO).

### Plasma and erythrocyte antioxidant activity

The effect of an onion-enriched diet on plasma antioxidant activity (ABTS^•+^ and FRAP values) and erythrocyte antioxidant enzyme (SOD and GPx) activities in hypercholesterolemic Wistar rats is summarised in Table 3. The HCO-fed group had higher ABTS^•+^ values compared to the control group and the HC-fed group. In concordance, the HCO-fed group had higher FRAP values compared to the HC-fed group. However, plasma ABTS^•+^ and FRAP values were not different between the control and the HC-fed groups. In contrast, SOD and GPx enzyme activities decreased significantly in HC-fed group compared to the control group. Nevertheless, those rats additionally fed with the functional ingredient maintained SOD activity in the same level as the control group, whereas GPx increased its activity compared to HC-fed group.

**Table 3:** **Plasma ABTS** ^**•+**^ **and FRAP values and erythrocyte antioxidant enzyme activities in rats fed the control, high-cholesterol (HC) and high-cholesterol enriched with onion (HCO) diets for 7 weeks**

	Control	HC	HCO
*Plasma*			
ABTS^•+^ (μmol TE/L)	6700.90 ± 72.76^a^	6634.29 ± 66.50^a^	6976.52 ± 39.96^b^
FRAP (μmol TE/L)	229.42 ± 15.71^ab^	201.22 ± 10.43^a^	258.96 ± 18.40^b^
*Erythrocyte*			
SOD (U/mL)	266.52 ± 27.66^b^	169.03 ± 15.16^a^	263.24 ± 22.78^b^
GPx (nmol/mL/min)	20.27 ± 2.46^c^	4.55 ± 0.70^a^	12.98 ± 0.87^b^

Values are expressed as the mean ± SEM (n = 8 rats per group).

HC, high-cholesterol diet; HCO, high-cholesterol enriched with onion diet.

Mean values within a row with unlike superscript letters were significantly different (*P* < 0.05). One-way ANOVA and posterior Tamhane's T2 and Bonferroni *post hoc* tests were used as appropriate.

### Plasma cholesterol and triacylglycerides levels

Dietary cholesterol content significantly affected plasma lipoprotein levels (Table 4). Rats fed the HC diet showed significantly higher plasma TC and LDL-C levels compared to the rats fed the control diet. Rats fed the HCO diet also showed significantly higher plasma TC and LDL-C levels compared to the rats fed the control diet. TC and LDL-C levels were not different between the HC- and HCO-fed groups. HDL-C levels were not different among the groups. The HC- and HCO-fed groups had higher atherogenic indexes compared to the control group. The HC- and HCO-fed groups had significantly lower TAG compared to the control group. TAG levels were not different between the HC- and HCO-fed groups.

**Table 4:** **Plasma total cholesterol, HDL-cholesterol, LDL-cholesterol, triacylglycerides and atherogenic indexes in rats fed the control, high-cholesterol (HC) and high-cholesterol enriched with onion (HCO) diets for 7 weeks**

	Control	HC	HCO
Total cholesterol (mg/dL)	83.88 ± 6.86^a^	198.25 ± 10.61^b^	224.88 ± 16.02^b^
HDL-cholesterol (mg/dL)	60.00 ± 4.94^a^	51.50 ± 3.74^a^	61.75 ± 4.12^a^
LDL-cholesterol (mg/dL)	11.88 ± 0.69^*a*^	118.63 ± 8.23^*b*^	123.50 ± 9.18^*b*^
AI (1)	0.205 ± 0.019^*a*^	2.398 ± 0.255^*b*^	2.083 ± 0.223^*b*^
AI (2)	1.405 ± 0.036^*a*^	3.959 ± 0.307^*b*^	3.711 ± 0.292^*b*^
AI (3)	0.405 ± 0.036^*a*^	2.959 ± 0.307^*b*^	2.711 ± 0.292^*b*^
Triacylglycerides (mg/dL)	84.38 ± 6.69^b^	40.50 ± 3.06^a^	32.63 ± 2.60^a^

Values are expressed as the mean ± SEM (n = 8 rats per group).

HC, high-cholesterol diet; HCO, high-cholesterol enriched with onion diet.

Mean values within a row with unlike superscript letters were significantly different (*P* < 0.05). One-way ANOVA and posterior Tamhane's T2 and Bonferroni *post hoc* tests were used as appropriate, italic small letters indicate Tamhane's T2 *post hoc* test.

AI, atherogenic index (1): LDL-cholesterol/HDL-cholesterol; AI (2): Total cholesterol/HDL-cholesterol; AI (3): (Total cholesterol-HDL-cholesterol)/HDL-cholesterol.

### Onion ingredient reverses endothelial dysfunction induced by high-cholesterol diet

**Figure 2:**
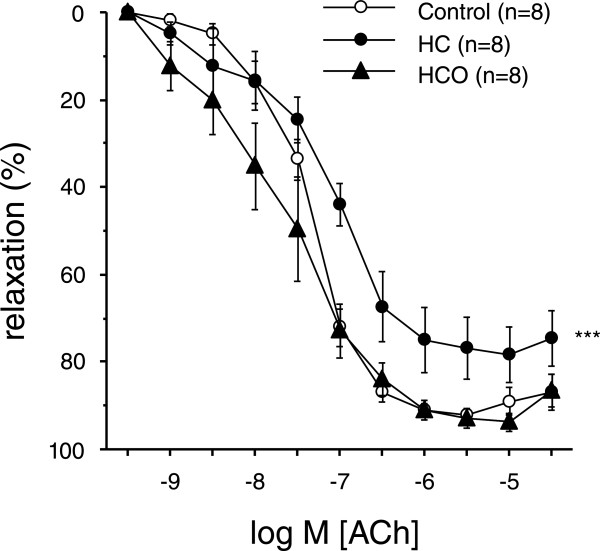
**Onion ingredient reverses endothelial dysfunction induced by high-cholesterol diet.** Endothelium-dependent relaxation induced by exposure to acetylcholine (ACh; 1 nM to 30 μM) in noradrenaline (NA)-contracted rat mesenteric arteries collected from rats fed the control, high-cholesterol (HC) and high-cholesterol enriched with onion (HCO) diets. Data are expressed as the mean ± SEM of the percentage of the remaining NA-induced contraction. n indicates the number of vascular segments used for the experiments obtained from 4–5 rats. ***P < 0.001 *vs*. control group by a two-way ANOVA test.

Acetylcholine (ACh; 1 nM to 30 μM) caused endothelium-dependent vasodilation in rat mesenteric arteries which was significantly impaired in rats fed with a HC diet (Figure 2). In contrast, endothelium-independent vasodilation induced by the NO donor, sodium nitroprusside (SNP; 1 to 100 μM) was not altered in HC-fed group and was not further enhanced by onion supplementation (E_max_: 90.5 ± 2.1%, 88.6 ± 5.4% and 78.1 ± 6.7% for control, HC-, and HCO-fed groups, respectively; n.s.). Then, it was evaluated the effects of onion enrichment on the alteration caused by HC diet on endothelial function. HCO diet completely prevented the impairment of endothelium-dependent vasodilation in mesenteric arteries from rats fed with a HC diet (Figure 2). There were no significant differences among the groups regarding the contraction induced by 120 mM K+ (12.6 ± 0.5 mN, 13.5 ± 1.1 mN and 13.6 ± 1.0 mN for control, HC-, and HCO-fed groups, respectively) and the contractile tone induced by noradrenaline to evaluate relaxation experiments (9.8 ± 0.8 mN, 11.8 ± 2.0 mN and 11.5 ± 0.8 mN for control, HC-, and HCO-fed groups, respectively).

### Increased superoxide generation from NADPH oxidase is responsible for high-cholesterol-induced endothelial dysfunction and is prevented by onion ingredient

**Figure 3:**
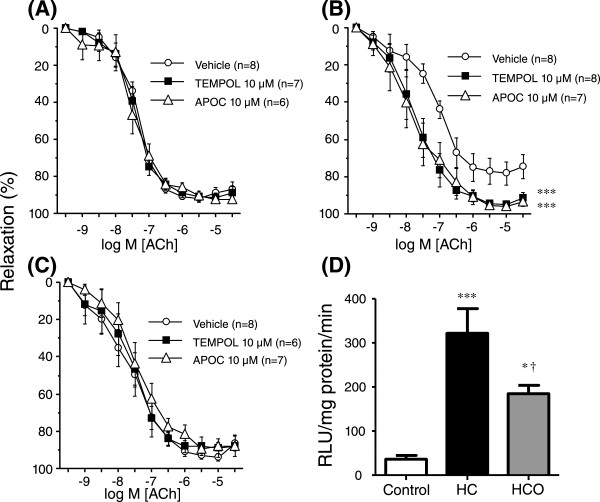
**Increased superoxide generation from NADPH oxidase is responsible for high-cholesterol-induced endothelial dysfunction and is prevented by onion ingredient.** Effects of the superoxide dismutase analogue, TEMPOL (10 μM), and the NADPH oxidase inhibitor, apocynin (APOC; 10 μM) on endothelium-dependent relaxation induced by exposure to acetylcholine (ACh; 1 nM to 30 μM) in noradrenaline (NA)-contracted rat mesenteric arteries collected from rats fed the control diet **(A)**, rats fed the high-cholesterol diet (HC) **(B)** and rats fed the high-cholesterol enriched with onion diet (HCO) **(C)**. Data are expressed as the mean ± SEM of the percentage of the remaining NA-induced contraction. n indicates the number of vascular segments used for the experiments obtained from 4–5 rats. ***P < 0.001 *vs*. control by a two-factors ANOVA test. **Panel D** shows determination of superoxide anions generated from NADPH oxidase by lucigenin-derived chemiluminiscence in mesenteric arteries collected from rats fed the control, HC and HCO diets. Data are expressed as mean ± SEM of relative light units (RLU)/mg of protein/min corresponding to 3 to 5 experiments. *P < 0.05, ***P < 0.001 *vs*. control group, †*P* < 0.05 *vs*. HC group by a one-way ANOVA followed by Student-Newman-Keuls test.

While scavenging of superoxide anions with the permeable superoxide dismutase mimetic, TEMPOL (10 μM), did not modify ACh-induced responses in mesenteric arteries from control group, the same treatment resulted in a significant improvement of endothelium-dependent vasodilation in mesenteric arteries from HC-fed group (Figure 3A and 3B). The improving effect induced by TEMPOL was absent in mesenteric arteries from HCO-fed group, in which evidence for endothelial dysfunction was not observed (Figure 3C). In fact, after TEMPOL treatment no differences in ACh-induced vasodilation were observed among the three groups (pD_2_ of 7.39 ± 0.10, 7.65 ± 0.22 and 7.59 ± 0.32 for control, HC-, and HCO-fed groups, respectively).

Since these results points to an increased generation of superoxide anions responsible for the endothelial dysfunction in HC-fed group, the role of NOX was investigated. Inhibition of NOX with apocynin (10 μM) did not modify ACh-induced vasodilation in control group, while a significant improvement of endothelial vasodilation in HC-fed group was observed, which was comparable in magnitude to that exerted by TEMPOL (Figure 3A and 3B). No effects induced by apocynin were observed in mesenteric arteries from HCO-fed group (Figure 3C). Analogously to that observed with TEMPOL, the differences in ACh-induced responses among the three groups of rats disappeared in the presence of the NOX inhibitor (pD_2_ of 7.46 ± 0.22, 7.58 ± 0.31 and 7.41 ± 0.20 for control, HC-, and HCO-fed groups, respectively; n.s.). Neither TEMPOL nor apocynin affected contractile tone induced by noradrenaline in mesenteric arteries from control (84.6 ± 6.4%, 84.9 ± 8.2% and 96.7 ± 5.5% of K^+^-induced contraction for vehicle, TEMPOL and apocynin, respectively; n.s.), HC-fed (87.6 ± 8.1%, 73.0 ± 7.1%, 79.1 ± 6.6% of K^+^-induced contraction for vehicle, TEMPOL and apocynin, respectively; n.s.), or HCO-fed groups (81.4 ± 6.5%, 84.7 ± 8.0%, 77.3 ± 6.6% of K^+^-induced contraction for vehicle, TEMPOL and apocynin, respectively; n.s.).

### Onion ingredient reduces the increment in NADPH oxidase activity induced by high-cholesterol diet in rat mesenteric arteries

To confirm the contribution of NOX to the increase in superoxide anion generation in mesenteric arteries from HC-fed rats suggested by the pharmacological inhibition of NOX in vascular reactivity studies, NOX activity in rat mesenteric arteries from the different groups was assessed. A significant increase in the NOX activity was observed in arteries obtained from HC-fed group, which was significantly reduced, but not abolished, in vessels from HCO-fed group (Figure 3D).

## Discussion

The present results evidence a clear positive effect of an onion-enriched diet on the preservation of the endothelium-dependent relaxation functions in the mesenteric arteries versus the impairment caused by a daily high-cholesterol intake (Figure 2). The development of the endothelial dysfunction is widely known to be influenced by several factors, such as the presence of insulin resistance, systemic inflammation, hypertension, or dysfunctional adipose tissue [30]. Obesity has been also associated with chronic inflammatory processes, atherosclerosis and oxidative stress, so constituting a pattern of microvascular dysfunction [31, 32]. Along with all these multiple disorders, the disruption of the metabolism of lipoproteins occurs involving an increased level of circulating LDL-C that, together with changes in the endothelial permeability, promote its entry and retention in the wall of the artery [33]. Consequently, it points out the hypercholesterolemia as an independent factor of risk for the progression of vascular dysfunction, atherosclerosis and cardiovascular disease [1, 34].

In this study the supplementation with cholesterol and cholic acid acting as a potential atherogenic diet was clearly reflected by the increased plasma TC and LDL-C levels. Nevertheless, no significant changes in HDL-C in HC- and HCO-fed groups, but a slight trend to decrease in HC-fed group compared to the control group, were appreciated. Atherogenic ratios [AI (1), AI (2) and AI (3) – Table 4] were unaffected between both HC- and HCO-fed groups, suggesting that the removal of cholesterol from the circulation was not enhanced by onion intake. However, several studies highlight a rise in TAG in HC-fed groups *vs*. control groups, while some of them show decreased levels of TAG in plasma or serum [35–38]. In this regard, the results in the present study showed decreased TAG levels after HC feeding (Table 4), thus differing from those studies where the hypercholesterolemia event (showing increased plasma TC and LDL-C, and decreased HDL-C levels) is simultaneously accompanied by an elevation of TAG and its reversion toward a basal status is achieved after feeding by specific diets (containing extracts or functional ingredients) or pharmacological interventions [39–43]. The concentration of TAG in the plasma at any time is generally the result of the balance between (I) the rate of entry of TAG into the plasma and (II) its rate of removal. In this sense, a possible cause of the cholesterol-induced hypotriglyceridemia may be the displacement of TAG by CE (cholesteryl esters) in newly formed VLDL particles. This mechanism has been recently described in Lxra−/− mice, accompanied by a decrease in lipogenic gene expression and a markedly reduced VLDL-TG production in cholesterol-fed animals [44]. Additionally, steatosis and hypotriglyceridemia in mice was reported by Werner *et al*. [45], concluding that this effect is a combined result of unimpaired hepatic TAG secretion, an increased hepatic synthesis of non-EFAs and the secretion of large VLDL particles, which may be subject to rapid clearance rates during EFA deficiency and development. Nonetheless, several mechanisms could be involved in this process since both cholesterol and bile acid metabolisms are involved in this tight regulation, as well as onion-enriched diet might be contributing to set the low TAG levels in the HCO-fed group, as a slight trend is appreciated when comparing HCO- and HC-fed groups [46, 47].

In addition to hypercholesterolemia, oxidative stress is known a major factor leading to endothelial dysfunction, with significant prognostic implications for cardiovascular events. Thus, enhanced reactive oxygen species (ROS) production by different isoforms of the NADPH oxidase present in the vascular wall are involved in vascular pathologies, such as hypertension, inflammation, atherosclerosis, and diabetic vasculopathy [48]. The contribution of onion antioxidants and related bioactive substances was expected to have some influence in the control of ROS production, depletion of antioxidant status, and NOX activity triggered by high dietary cholesterol. According to our previous hypothesis, the findings in this study on plasma antioxidant activity evidenced significantly higher ABTS^•+^ and FRAP plasma values in rats fed the HCO diet compared to those fed the control diet. This fact was in line with the preventive effect achieved in superoxide anions generation promoted by NOX, which confirmed that onion supplementation can reduce the exacerbated NOX activity mediating endothelial dysfunction in the mesenteric arteries from the hypercholesterolemic animals (Figure 2). In addition, the consumption of onion as functional ingredient prevented the depletion of SOD and GPx enzyme activities, suggesting also a higher capacity of HCO fed-group by detoxifying ROS, in agreement with recent studies [49, 50].

The vascular-protective role of flavonoids (and especially their antioxidant properties) is an active field under investigation to improve the endothelial function through the modification of the oxidative stress status [51]. The onion powder used in the current study presented quercetin and isorhamnetin derivatives as major flavonols on its composition (Table 1), highlighting that a preventive function on AngII-induced endothelial dysfunction and an increased response to NO can be achievable with both compounds too [52]. Moreover, Shen *et al*. [53] have recently shown how the supplementation of quercetin is able to induct heme oxygenase-1 (HO-1) protein expression, protecting endothelial cells against oxidative damage. Among other factors such as a reduced ERK1/2 phosphorylation, a decreased NFκB activation and a down-regulation of the Ob-Ra expression [54] or the inhibition of pro-inflammatory cytokines [55, 56] that have proved the anti-inflammatory effects exerted by quercetin.

Furthermore, interesting results have been described for raw onion and onion extracts in relation to the inhibition of platelet aggregation [57], anti-apoptotic activity in DOX-mediated entothelial cells [58], antioxidant protection against lipoprotein oxidation and oxidative stress [47, 59], and regulation of endogenous H_2_S pathway [60, 61].

The mechanisms of action by which onion can enhance vasodilatory responses are multiple. McNeill and Jurgens [62] reported the implication of the endothelium-dependent vasodilatation, nitric oxide and cGMP mediated vasodilation in response to Welsh onion extract in the aorta of rats. The improving effects of HCO diet on endothelial vasodilation are not related to a potentiation of NO/cGMP pathway-mediated relaxation of smooth muscle since HCO diet did not enhance relaxant capacity of SNP beyond a preserved response in mesenteric arteries from HC-fed rats. In the present work, the hypercholesterolemia induced by cholesterol supplementation has been clearly associated with the imbalance status achieved in mesenteric arteries in Wistar rats, by forcing endothelial dysfunction, affecting endothelium-dependent vascular smooth muscle relaxing capability, as well as involving NOX stimulation. On the other hand, the intake of the onion as functional ingredient during seven weeks evidenced an improvement and total reversion of such negative effects, specifically in response to cumulative dose of ACh. It has been suggested that apocynin could act as an antioxidant rather than a NOX inhibitor in vascular cells because of the lack of myeloperoxidase (MPO) activity that converts apocynin into its radical form that generates active dimers [63]. However, in addition to the fact that non-MPO peroxidases could account for the formation of apocynin dimers and trimers in vascular tissue [64], in the present study culture cells are not being evaluated, but a complete arterial segment that could easily contain MPO activity, mainly in hypercholesterolemic animals since hypercholesterolemia facilitates MPO activity increase in vascular tissues [65, 66]. Thus, although the antioxidant capacity of apocynin could account for its reversing effects on hypercholesterolemia-associated endothelial dysfunction, it would be reasonable to consider that such effects are contributed by the ability of apocynin to inhibit NOX. The involvement of increased NOX activity in the impairment of endothelial vasodilation caused by hypercholesterolemia is supported by the fact that elevated NOX activity is detected in mesenteric arteries from HC-fed rats. This suggests that onion metabolites (or the changes that they may induce in metabolism) may partially reduce the increased NOX activity in the hypercholesterolemic rats, which can help to reduce the risk of some pathologies like CVD to a certain extent. Although a direct inhibitory effect on vascular NOX activity by onion metabolites cannot be discarded, the enhancement of the plasma antioxidant activity likely reflects a systemic reduction of the prooxidant and pro-inflammatory status in these animals. However, other studies regarding the contribution of eicosanoids products, the role of K+ and calcium channel, or EDHF, among others [67], should be further explored to know all mechanisms of action contributing to vasodilation in the vasculature of rats after the onion consumption.

Additionally, as the underlying mechanisms involved in these antiatherosclerotic effects of onion and prevention of CVD are complex and not well known yet, several factors must remain under study and discussion, as for example the synergic implication of sulphur-containing compounds, which can be transformed chemically or enzymatically in the organism with their subsequent formation of H_2_S. Therefore, the implication of sulfides and other sulphur substances, besides other bioactive compounds found in the onion powder are assumed to contribute in the reversion of vascular impairment found in endothelium-dependent vasodilation in the mesenteric vasculature and the enhanced antioxidant status found in the HCO-fed group.

## Conclusions

Since the search for modifications in diet and lifestyle which contribute to the reversion of endothelial dysfunction remains active (e.g. lipid lowering-therapy, n3 fatty acids, vitamin C, folate, etc.) [30, 68]; the enrichment of diet with onion ingredients may be discussed as a complementary strategy to prevent or partially modulate vascular dysfunction, reducing some of the risk indexes linked to initial stages of atherosclerosis.

Further investigation is needed to improve the understanding of the mechanisms of action and the implications of onion in the prevention and development of cardiovascular and metabolic diseases, to finally dissect its effects and its possible functions and interactions with other functional compounds.
